# The Effect of Coenzyme Q10 on Tinnitus Severity and Sleep Quality in Patients with Presbycusis

**DOI:** 10.22038/ijorl.2024.79602.3681

**Published:** 2025

**Authors:** Rohollah Abbasi, Faranak Emami, Saeid Atighechi, Zahra Sadeghi

**Affiliations:** 1 *Department of Otolaryngology Head and Neck Surgery, School of Medicine, Hamadan University of Medical Sciences, Hamadan, Iran. *; 2 *Department of Audiology, School of Rehabilitation, Hearing Disorders Research Center, Hamadan University of Medical Sciences, Hamadan, Iran.*; 3 *Otorhinolaryngology Research Center, Shahid Sadoughi University of Medical Sciences, Yazd, Iran.*

**Keywords:** Coenzyme Q10, Presbycusis, Tinnitus

## Abstract

**Introduction:**

Tinnitus is one of the symptoms of presbycusis that affects patients' sleep and social life. This study aimed to determine the effect of coenzyme Q10 (CoQ10) on treating tinnitus due to presbycusis.

**Materials and Methods:**

In this double-blind, randomized clinical trial, 50 patients with tinnitus due to presbycusis were randomly divided into groups A and B, with 25 patients in each group. In addition to routine treatments, group A received 100 mg of CoQ10 daily, while group B received a placebo. Both groups were evaluated for tinnitus severity, loudness of tinnitus, quality of life, and sleep disturbance before and 6 weeks after starting the treatment.

**Results:**

In the intervention and control groups, the mean changes in score compared to before the treatment were as follows: quality of life (3.1 ± 1.67) and (1.28 ± 0.76) (P = 0.298), sleep disorder (-7.60 ± 1.38) and (-1.0 ± 8.55) (P<0.001), tinnitus disability (-17.2 ± 52.93) and (-4.56 ± 1.37) (P<0.001), tinnitus loudness of right ear (-1.68 ± 0.41) and (-0.95 ± 0.23) (P=0.11) and left ear (-2.2 ± 0.35) and (-0.54 ± 0.21) (P<0.001).

**Conclusion:**

This study indicated that adding CoQ10 to the routine regimen for patients with tinnitus due to presbycusis significantly decreases tinnitus disability, improves sleep disturbance, and reduces tinnitus loudness. However, more studies should be conducted in this regard.

## Introduction

Presbycusis refers to hearing loss due to aging (1). There are a variety of factors affecting presbycusis. This condition involves bilateral and symmetrical lesions, characterized by slow and progressive changes, often resulting in high-frequency hearing loss (2). Presbycusis affects social life (3-5). Tinnitus is one of the symptoms of presbycusis (6). Tinnitus refers to the perception of repetitive sound, often ringing in one or both ears without any stimulus (7). It is a very common complaint among those who visit otolaryngologists. Tinnitus is the perception of sound without an external source, often heard as ringing, buzzing, or snake noises in one or both ears. According to some studies, 10-15% of the population suffers from tinnitus (8). It affects 30% of people over 55, with an annual incidence of 5%. About 50 million people in the United States suffer from tinnitus (9), with bothersome tinnitus occurring in 3-5% of these patients (10). 

Tinnitus is sometimes associated with hearing loss, depression, anxiety, restlessness, sleep disorders, and other mood disorders (11). No definitive and specific treatment for tinnitus has been reported so far. Current treatments include hearing aids, sound therapy, ambient sound enrichment, supportive therapies, and vasodilators using corticosteroids, anticonvulsants, antispasmodics, lidocaine, benzodiazepines, and others. These have often failed due to different etiologies of tinnitus and unknown mechanisms (12).

Coenzyme Q10 (CoQ10) is a major antioxidant from the ubiquinone family naturally produced in the body. This fat-soluble compound, which plays an important role in energy production, is found in large amounts in the mitochondria of cells in the heart, liver, immune system, and kidneys that require much energy. Its highest concentration is in the heart, liver, kidneys, and pancreas tissues, which are involved in various essential processes in the body. Besides, its effect on the mitochondrial electron transfer chain is well known. This enzyme also seems to have membrane-stabilizing properties and is an antioxidant associated with vitamin E (13). CoQ10 has been used as adjuvant therapy in many diseases, such as chronic heart failure, and it has also been reported to be effective in the treatment of hypertension, muscular dystrophy, and neurodegenerative diseases (14). Under normal conditions, the body's cells can supply the required amount without external support. In cases of deficiency of this enzyme, external supplementation can lead to higher concentrations in the cell membrane. The main food sources of CoQ10 are red meat, poultry, fish, liver, and certain vegetables (15). In age-related degeneration and neurodegenerative diseases, such as Parkinson's disease, low levels of coenzyme CoQ10 have led to the belief that it plays a role in oxidative damage caused by reactive oxygen species (16). CoQ10 administration has been relatively effective in patients with Parkinson's disease (17). It has also been shown to protectagainst ischemic damage induced by mitochondrial toxins (18). Coenzyme Q10 is a potent antioxidant that removes free radicals directly or by participating in the recovery cycle of other antioxidants (19, 20). The relationship between psychological stress and tinnitus has been demonstrated in some studies (21). Tinnitus therapy remains a challenge in ENT medicine. Many studies have been conducted to find the most effective therapy with minimal side effects, often yielding contradictory results. Therefore, this research was designed to investigate the effect of CoQ10 on tinnitus severity and sleep quality in patients with presbycusis.

## Materials and Methods

After obtaining approval from the local ethics committee and written informed consent, this double-blind, randomized clinical trial involved 50 patients with tinnitus due to presbycusis. Inclusion criteria were tinnitus lasting at least six months, a THI score above 10, and bilateral sensorineural hearing loss. Exclusion criteria included psychosomatic disorders, calcium, vitamins, or magnesium intake two months before and during the intervention, tinnitus due to drug complications, atypical tinnitus, Meniere's disease, conductive hearing loss, and any sensitivity or intolerance to the drug. A psychiatrist initially examined Patients to rule out psychosomatic disorders, and then an otolaryngologist for cerumen accumulation and acute or chronic ear infections. Pure tone audiometry (PTA), speech reception threshold (SRT), speech discrimination scores (SDS), and tympanometry were performed on all patients. The tinnitus matching test (TMT), consisting of frequency determination and loudness of tinnitus, was also conducted. The same audiologist performed all tests. The Pittsburgh Insomnia Rating Scale, Tinnitus Handicap Inventory (THI), and the Quality of Life Questionnaire (SF-36) were completed by patients in the presence of an audiologist. 

Patients were randomly assigned to treatment groups A and B using a random number table. Both groups received routine therapy consisting of 25 mg/day of Nortriptyline for six weeks (15). The intervention group took 100 mg of CoQ10 daily for six weeks, while the control group received a placebo. After the treatment period, audiometry was repeated, and a blinded evaluator reassessed patients for sleep disorders (Pittsburgh test), tinnitus severity (THI), and quality of life (SF-36). In this study, the medications were packaged with codes A and B. The prescribing doctor, patient, and evaluator were all unaware of the contents of the codes until after data analysis (double-blind study). The relevant codes were revealed only after data analysis. Due to randomization, the groups were similar in terms of age, sex, and other potential confounding variables. The questionnaire data were entered into SPSS-16 after completion. The Chi-squared and Fisher's exact tests were used to compare nominal qualitative variables. Given the small sample size, the mean scores were compared using the Student's t-test in both groups to compare the THI scores for sleep disorders and quality of life. A P-value < 0.05 was considered statistically significant.

## Results

In this study, the patients in the intervention and control groups were similar in age, sex, duration of disease, and side of ear involvement (Table 1). 

**Table 1:** Basic information of the examined patients

**Variable**	**Group**		**P- value**
	**Intervention** **(n=25)**	**Control(n=25** **)**	
Sex	N(%)	N(%)	
Male Female	(56.0)14 (44.0)11	(60.0)15	0.774*
Ear	N(%)	N(%)	
Unilateral Bilateral	(56.0)14 (44.0)11	(64.0)15 (36.0)10	0.584*
Hearing loss	N(%)	N(%)	
Yes No	(100)25 (0)0	(100)25 (0)0	-
Age	Mean ± SD	Mean ± SD	
Year	52.67±12.11	50.20±11.97	0.447**
Duration of disease	Mean ± SD	Mean ± SD	
Month	15.12±14.82	18.52±18.07	0.480**

* Chi-squared test

** Mann–Whitney U test

No statistically significant difference was observed between the intervention and control groups in terms of the mean quality of life score before and after the treatment. However, the mean score for tinnitus disability and sleep disorder in the intervention group was significantly reduced compared to the control group (Table 2).

**Table 2:** Frequency of sleep score disorder, tinnitus disability, and quality of life before and after the intervention in the intervention and control groups

**Variable**	**Group**			P- value*
	**Intervention Mean ± SE**		**Control Mean ± SE**	
Quality of life (SF36)				
Score difference After-before	3.1 ± 24.67	1.0 ± 28.76		0.298
Pediatric Sleep Questionnaire (PSQ)				
Score difference After-before	-7.1 ± 60.38	-1.0 ± 08.55		<0.001
Tinnitus Handicap Inventory (THI)				
Score difference After-before	-17.2 ± 52.93	-4.1 ± 56.37		<0.001

The loudness of tinnitus in the left ear of the patients in the intervention group was significantly reduced compared to the control group (Table. 3).

**Table 3:** Comparison of the mean score for tinnitus loudness in the intervention and control groups

**Assessment time**	**Group**		P-value*
	**Intervention** **Mean ± SD**	**Control** **Mean ± SD**	
Right ear			
After-before	-1.0 ± 68.41	-0.0 ± 95.23	0.117
Left ear			
After-before	-2.0 ± 20.35	-0.0 ± 54.21	<0.001

## Discussion

Since tinnitus results from the interaction between the auditory and central nervous systems, it may be a stressor, potentially leading to psychological and subsequent oxidative stress (14). Antioxidant therapy, such as CoQ10 (22), may help reduce oxidative stress and inner ear damage in patients with idiopathic tinnitus, alleviating the intensity and discomfort associated with the condition (23). 

In this study, the intake of CoQ10 significantly reduced tinnitus disability and loudness. However, very few studies have explored the relationship between CoQ10, sleep disorders, and tinnitus severity. Khan et al. (2007) conducted a study titled "Effect of CoQ10 on Chronic Tinnitus in 20 Patients," which found that CoQ10 administration improved tinnitus symptoms in patients with low plasma CoQ10 levels (14). Our study included a larger sample size, and although we did not measure plasma CoQ10 levels, our findings are consistent with Khan's in demonstrating the beneficial effects of CoQ10 on tinnitus. This alignment suggests that CoQ10 may be broadly effective in reducing tinnitus symptoms, regardless of baseline plasma levels, though further research could clarify this relationship. Additionally, a case-control study by Scasso et al. (2017) explored the effect of antioxidant supplementation, including CoQ10, on preventing auditory complications like tinnitus in patients undergoing cisplatin chemotherapy. Their findings indicated oral CoQ10 administration could prevent cisplatin-induced hearing problems, including tinnitus (24). In contrast, our study focused on the therapeutic effects of CoQ10 on existing tinnitus, rather than its preventive potential. Despite this difference in focus, both studies highlight CoQ10's efficacy in mitigating tinnitus symptoms as a preventative or therapeutic measure. Similarly, Staffa et al. (2014) assessed the effect of CoQ10 on the recovery time for noise-induced hearing loss and residual tinnitus. Their study indicated that CoQ10 treatment leads to faster recovery, likely due to its therapeutic effect on hair cells in response to oxidative stress. 

While Staffa's research focused on hearing loss, our study specifically investigated CoQ10's impact on chronic tinnitus and found that, in addition to improving hearing loss, CoQ10 also reduced tinnitus symptoms (25). Overall, our findings add to the growing body of evidence supporting CoQ10's effectiveness in reducing tinnitus symptoms. The consistency of results in various studies, despite differences in patient populations, conditions (e.g., chemotherapy-induced hearing problems, noise-induced hearing loss), and research designs, highlights CoQ10's potential as a therapeutic agent for tinnitus. Notably, our study focused specifically on chronic tinnitus, thereby expanding the application of CoQ10 beyond preventive care or recovery from acute auditory damage. Further research, especially studies that measure plasma CoQ10 levels and explore its relationship with sleep disorders and tinnitus severity, would help to refine our understanding of the mechanisms behind CoQ10's effectiveness and identify which patient groups might benefit most from this treatment. In our study, the daily intake of 100 mg of CoQ10 for six weeks did not significantly affect patients' quality of life. It is consistent with findings from a study by Polanski et al. (2016) in Brazil, titled "Effect of antioxidant therapy on adult tinnitus disability," where the tinnitus handicap inventory (THI) was used to assess disability before and 60 months after treatment. Polanski et al. found no statistically significant difference in THI scores before and after treatment (26). 

Our results align with Polanski's, although our study focused on the effects of CoQ10 specifically, whereas Polanski's evaluated other antioxidants. Furthermore, CoQ10 possesses anti-inflammatory properties, which may help reduce inflammation by targeting pro-inflammatory cytokines like IL-6 and TNF-α, potentially contributing to the alleviation of tinnitus symptoms (27). In the present study, CoQ10 intake significantly improved sleep disorders in patients with tinnitus due to presbycusis. CoQ10 positively affects auditory hair cells, reduces fatigue, and enhances sleep quality. Based on the results of various studies, CoQ10 has been observed to improve sleep by reducing patient fatigue (28-30). 

The beneficial effects of CoQ10 on sleep were demonstrated by Gvozdjáková et al. in a study involving 24 children, in which ubiquinol therapy improved sleep issues in 34% of the participants (31). More recently, Mousavinejad et al., found that high doses of CoQ10 significantly improved subjectively reported sleep disorders compared to placebo and low-dose treatments (32). CoQ10's mechanism of action seems linked to its role in intracellular energy production, the mitochondrial electron-transport chain, and ATP synthesis within the mitochondrial membrane (22). 

Additionally, CoQ10 is recognized as a safe and effective supplement for reducing fatigue symptoms (33). Thus, CoQ10 may enhance sleep through energy regulation and alleviating stress and fatigue. Another researcher suggested that exogenous CoQ10 supplementation can help prevent stress or adverse biochemical changes associated with exercise-related energy depletion (22). It indicates that CoQ10's benefits might extend beyond sleep improvement, including stress reduction and overall energy regulation.

*Strengths of the study*

The results are important since no similar study has evaluated the effect of this intervention on sleep quality and tinnitus severity in patients with presbycusis. In addition, the groups were similar in age, sex, and other potential confounding variables in this study.

*Limitation of study*

- Sample Size: The study included a relatively small sample size of 50 participants, which may limit the generalizability of the findings. Larger studies are needed to confirm these results and establish broader applicability.
- **Single Dosage**: Only one dosage of CoQ10 (100 mg/day) was tested. Exploring different dosages could help determine the optimal amount of CoQ10 required to achieve the best therapeutic effects.
- **Lack of Plasma CoQ10 Measurement**: The study did not measure plasma CoQ10 levels before and after treatment, which could have provided valuable information about the correlation between plasma levels and clinical outcomes.
- Outcome Measures: The study uses subjective measures like tinnitus severity, quality of life, and sleep disturbance, which can be influenced by individual perception and reporting bias. Additionally, how these were measured (e.g., through self-reported questionnaires) can affect the reliability of the results.

## Conclusion

According to the present study, it seems that adding coenzyme Q10 to the routine regimen for patients with tinnitus due to presbycusis significantly decreases tinnitus disability, improves sleep disturbance, and reduces tinnitus loudness. However, it may not significantly impact the overall quality of life. In addition, the effect of coenzyme Q10 was greater in men over 50, and the duration of infection was less than a year. However, it is suggested that more studies be conducted in this regard.

## Recommendations

In future studies with a larger sample size, it is suggested to examine the mechanism of the effect of CoQ10 on tinnitus due to presbycusis, particularly in sex and age differences.
